# Elevated NLRP3 Inflammasome Activation Is Associated with Motor Neuron Degeneration in ALS

**DOI:** 10.3390/cells13120995

**Published:** 2024-06-07

**Authors:** Hilal Cihankaya, Verian Bader, Konstanze F. Winklhofer, Matthias Vorgerd, Johann Matschke, Sarah Stahlke, Carsten Theiss, Veronika Matschke

**Affiliations:** 1 Department of Cytology, Institute of Anatomy, Ruhr-University Bochum, 44801 Bochum, Germany; hilalcihankaya@gmail.com (H.C.); carsten.theiss@rub.de (C.T.); 2 International Graduate School of Neuroscience (IGSN), Ruhr-University Bochum, 44801 Bochum, Germany; konstanze.winklhofer@rub.de; 3 Department of Molecular Cell Biology, Institute of Biochemistry and Pathobiochemistry, Medical Faculty, Ruhr-University Bochum, 44801 Bochum, Germany; verian.bader@rub.de; 4 Department of Neurology, Heimer Institute for Muscle Research, University Hospital Bergmannsheil, Ruhr-University Bochum, Buerkle-de-la-Camp-Platz 1, 44789 Bochum, Germany; matthias.vorgerd@bergmannsheil.de; 5 Institute of Cell Biology (Cancer Research), University Hospital Essen, University of Duisburg-Essen, 45147 Essen, Germany; johann.matschke@uk-essen.de

**Keywords:** amyotrophic lateral sclerosis, motor neuron degeneration, NLRP3 inflammasome, miR-223-3p, pyroptotic cell death, wobbler mouse

## Abstract

Amyotrophic lateral sclerosis (ALS) is a fatal neurodegenerative disease characterized by motor neuron degeneration in the central nervous system. Recent research has increasingly linked the activation of nucleotide oligomerization domain-like receptor protein 3 (NLRP3) inflammasome to ALS pathogenesis. NLRP3 activation triggers Caspase 1 (CASP 1) auto-activation, leading to the cleavage of Gasdermin D (GSDMD) and pore formation on the cellular membrane. This process facilitates cytokine secretion and ultimately results in pyroptotic cell death, highlighting the complex interplay of inflammation and neurodegeneration in ALS. This study aimed to characterize the NLRP3 inflammasome components and their colocalization with cellular markers using the wobbler mouse as an ALS animal model. Firstly, we checked the levels of miR-223-3p because of its association with NLRP3 inflammasome activity. The wobbler mice showed an increased expression of miR-223-3p in the ventral horn, spinal cord, and cerebellum tissues. Next, increased levels of NLRP3, pro-CASP 1, cleaved CASP 1 (c-CASP 1), full-length GSDMD, and cleaved GDSMD revealed NLRP3 inflammasome activation in wobbler spinal cords, but not in the cerebellum. Furthermore, we investigated the colocalization of the aforementioned proteins with neurons, microglia, and astrocyte markers in the spinal cord tissue. Evidently, the wobbler mice displayed microgliosis, astrogliosis, and motor neuron degeneration in this tissue. Additionally, we showed the upregulation of protein levels and the colocalization of NLRP3, c-CASP1, and GSDMD in neurons, as well as in microglia and astrocytes. Overall, this study demonstrated the involvement of NLRP3 inflammasome activation and pyroptotic cell death in the spinal cord tissue of wobbler mice, which could further exacerbate the motor neuron degeneration and neuroinflammation in this ALS mouse model.

## 1. Introduction

Amyotrophic lateral sclerosis (ALS) is a fatal neurodegenerative disease, characterized by rapid progressive degeneration of the upper motor neurons in the motor cortex and the lower motor neurons in the spinal cord and brain stem. The incidence ranges from 0.26 to 23.46 per 100,000 people per year worldwide [1], and mostly older men rather than women are affected [2]. The clinical symptoms and the clinical course of the disease depend largely on which motor neurons in the central nervous system are affected. Generally, ALS patients suffer from muscle weakness, muscle atrophy, hyperreflexia, spasticity, and paralysis [3]. Ultimately, patients die due to respiratory failure within 1–5 years after the onset of disease symptoms [3]. In most cases, the etiology of ALS remains unknown. The most common form of ALS is sporadic ALS (sALS), which occurs in 90–95% of patients and develops without a genetic background. The second form of the disease is familial ALS (fALS), which is seen in only 5–10% of patients and inherited in a genetically dominant way. Currently, there has been no effective treatment for ALS; however, several drugs are available to improve the symptoms of ALS patients. The ongoing need for more research to find more effective treatments and to understand the molecular mechanisms underlying ALS has led to the use of experimental animals.

The wobbler mouse, exhibiting motor neuron degeneration, is the only naturally occurring sALS animal model in use today, since it emerged spontaneously in the laboratory [4]. Similar to ALS patients, wobbler mice show motor defects, tremors, muscle weakness, cramps, hyperreflexia, and finally, die due to respiratory failure [5]. The leading cause of all these abnormalities was found to be an autosomal recessive point mutation in the vacuolar protein sorting-associated protein 54 (*Vps54*) gene in 2005 by Schmidt-John et al. [6]. VPS54 protein is one of the subunits of the Golgi-associated retrograde protein (GARP) complex, and it takes the role of the retrograde vesicular transport of molecules from early/late endosomes to recycling endosomes and the trans-Golgi network. The so-called wobbler mutation first destabilizes the VPS54 protein, which in turn destabilizes the GARP complex, leading to impairments in retrograde vesicular transport. However, the precise mechanisms showing how the wobbler mutation is linked to ALS are still being investigated.

Motor neuron degeneration is generally accompanied by neuroinflammation in both ALS patients [7,8] and in the animal models of ALS [9]. Neuroinflammation is the natural response of the innate immune system in the central nervous system (CNS), and when left uncontrolled, chronic inflammation starts to pose a danger to motor neurons, causing premature cell death. The activation of neuroinflammatory reactions in the CNS can be triggered by pathogen-associated molecular patterns (PAMPs) and damage-associated molecular patterns (DAMPs), which can be recognized by pattern recognition receptors (PRRs) [10]. Of these, the nucleotide oligomerization domain-like receptor protein 3 (NLRP3) inflammasome, an intracellular sensory multiprotein complex, has recently attracted considerable attention. The NLRP3 inflammasome is formed by a sensor protein NLRP3, an adaptor protein apoptosis-associated speck-like protein (ASC), and an effector protein Caspase 1 (CASP 1) [11]. Upon activation either by DAMPs or PAMPs, due to increased levels of ROS or mitochondrial damage, pro-CASP 1 undergoes auto-catalytic cleavage, leading to the secretion of IL-1β and IL-18. Furthermore, activated CASP-1 induces the cleavage of the Gasdermin D (GSDMD) protein; therefore, N-terminal-GSDMD proteins translocate into the membrane to form pores, which ultimately causes pyroptotic cell death. Pyroptotic cell death is an inflammatory form of programmed cell death, which is induced by the activation of Caspase 1 or 11 in mice, and 1, 4, or 5 in humans [12,13]. It is characterized by pore formation on the cell membrane, cell swelling, membrane rupture, and ultimately the leakage of cytosolic contents into the extracellular space [14].

The regulation of NLRP3 inflammasome activation can occur at the transcriptional or post-translational level. miRNAs are one of the post-transcriptional regulatory molecules that modulate the activation of the NLRP3 inflammasome. Of these, miR-223-3p has been shown to regulate the NLRP3 inflammasome in several neurodegenerative diseases [15]. Specifically, miR-223-3p suppresses NLRP3 expression by binding to its 3′-UTR, reducing activation and the release of inflammatory cytokines like IL-1β and IL-18 [16,17]. Studies show its overexpression attenuates inflammatory responses in diseases such as rheumatoid arthritis [18] and Crohn’s disease [19]. The dysregulation of miR-223-3p is also observed in neurodegenerative disease models like ALS [20] and multiple sclerosis [15], highlighting its role in CNS inflammation. However, miR-223-3p can also have pro-inflammatory effects [17]. For example, miR-223-3p was found to be upregulated in patients with Alzheimer’s disease [21], dementia [21], Parkinson’s disease [22], Huntington’s disease [23], epilepsy [24], age-related macular degeneration [25], and ataxia [26]. In some models, its upregulation enhances inflammation and promotes the differentiation of pro-inflammatory macrophages [27]. This dual role may depend on cellular context and specific molecular targets [28]. While miR-223-3p generally acts as an anti-inflammatory agent, it can also activate pro-inflammatory pathways like NFκB under certain conditions [29,30].

Given the complex role of miR-223-3p in inflammation and its potential impact on NLRP3 inflammasome activity, we hypothesized that NLRP3 inflammasome activation and pyroptotic cell death might be involved in the motor neuron degeneration observed in ALS. Having shown increased levels of ROS [31,32] and mitochondrial damage [33], we aimed to investigate the expression, activation, and localization of NLRP3 inflammasome components in the spinal cord of the wobbler mouse. Therefore, in this study, we explored pyroptotic cell death as a cellular mechanism underlying motor neuron degeneration in ALS using the wobbler mouse as an animal model.

## 2. Materials and Methods

### 2.1. Animals

All protocols for animal experiments were approved by the German federal state of North Rhine Westphalia in accordance with the European Communities Council Directive 2010/63/EU on the protection of animals used for scientific purposes (Registration number Az. 84-02.04.2017.A085). Animals were kept at constant room temperature (22 °C) on a 12:12 light–dark cycle. Food and water were supplied ad libitum. The breeding, handling, and genotyping of mice were carried out as previously described [34]. For the experiments, cervical spinal cord and cerebellum tissues from both wild-type (WT) and wobbler (WR) mice were collected at p40, and heterozygous mice were used for breeding. Animals from both sexes were used in this study since disease onset and progression have not been associated with gender [35].

### 2.2. Cresyl Violet Staining and Laser Microdissection (LMD) Microscope

Since the wobbler mouse shows motor neuron degeneration, we were interested in the gray matter of the ventral horn, where motor neurons are mainly located. Additionally, we used the cerebellum as a negative control, as neuronal cell death in the cerebellum of wobbler mice has not been reported in the literature. Cresyl violet staining was used to detect motor neurons and to separate white/gray matter and dorsal/ventral horns in the cervical spinal cord sections.

Wild-type and wobbler mice were decapitated, and the cervical parts of the spinal cords were collected. Then, tissues were deeply frozen in isopentane at −45 °C for 5–10 s and kept at −80 °C until further use.

Then, 20 µm spinal cord sections from wild-type and wobbler mice were sliced using cryostat, and sections were mounted on RNase-free PET membrane slides (Leica, 11505190, Wetzlar, Germany). For the staining protocol, slices were treated with 70% ethanol in DEPC water at 4 °C for 2 min. Then, tissues were stained with 1% cresyl violet (Sigma-Aldrich, 61135-25G, St Louis, MO, USA) in DEPC-treated water for 1 min. Next, slices were washed with 70% ethanol in DEPC water at room temperature, and finally, they were dehydrated by a 1 s treatment of 100% ethanol at 4 °C.

**Figure 1:**
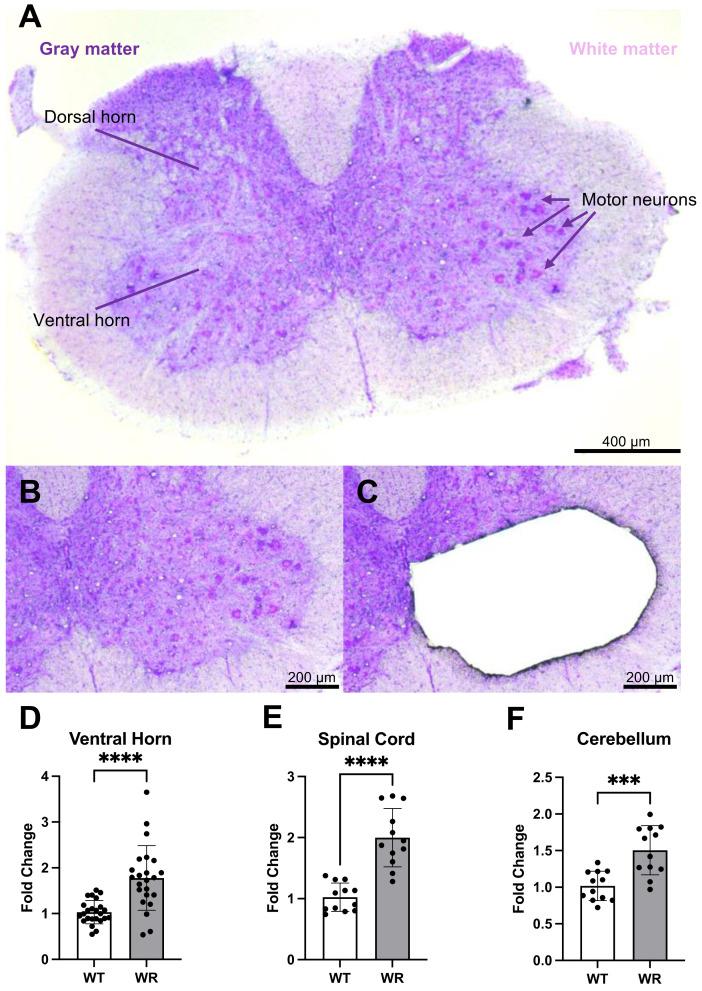
Wobbler mice demonstrated elevated levels of miR-223-3p in laser-microdissected ventral horn, cervical spinal cord, and cerebellum. (**A**) Image of cresyl-violet-stained spinal cord section. Gray matter can be seen as dark purple, and white matter can be seen as light purple/pink. Blunt-ended lines show ventral and dorsal horns. Arrows show individual motor neurons. Scale: 400 µm. Close-up image of cresyl-violet-stained gray matter of ventral horn (**B**) before and (**C**) after LMD. Scale: 200 µm. RT-qPCR analysis of miR-223-3p using samples from (**D**) laser-microdissected ventral horn, (**E**) cervical spinal cord, and (**F**) cerebellum of p40 wild-type and wobbler mice. The differences in the expression levels were calculated using the 2^−ΔΔCt^ method with endogenous normalization to UniSp6. Data are presented as mean ± SD, n = 4–8. *** *p* < 0.001; **** *p* < 0.0001.

Cresyl-violet-stained gray matter of ventral horns on the RNAse-free PET membranes was lasered using an LMD microscope (Leica, LMD6000 system, Wetzlar, Germany), as shown in Figure 1. Lasered samples were collected in 2 sessions/sample at the lids of non-adhesive 0.5 mL tubes, containing 50 µL lysis solution (AM1931, Thermo Fisher Scientific, Waltham, MA, USA). Then, ~27 mm^2^ lasered area per mouse was collected by LMD directly into the lysis solution. After collection, samples were kept at −80 °C until further use.

### 2.3. miRNA Isolation

miRNA isolation of the lasered WT and WR samples was performed using an RNAqueous-Micro Total RNA Isolation Kit based on the manufacturer’s instructions (Thermo Fisher Scientific, AM1931, Waltham, MA, USA). Optional DNase I treatment and DNase inactivation steps were not performed. RNA concentrations were measured by Nanodrop One C (Thermo Scientific, Waltham, MA, USA), and on average, ~28 µg/µL RNA in 16 µL DEPC-treated water was obtained. Samples were kept at −80 °C until further use.

In addition to the lasered samples, miRNA isolation from the cerebellum and cervical spinal cord of WT and WR mice was performed using the Nucleo-Spin miRNA Kit (Macherey-Nagel, 740971, Düren, Germany) based on the manufacturer’s instructions. Total RNAs (tRNAs) were eluted in 30 µL RNase-free water, and RNA concentrations were measured using Nanodrop One C (Thermo Scientific, Waltham, MA, USA). Samples were kept at −80 °C until further use.

### 2.4. RT-PCR and qPCR

To obtain cDNA from the WT and WR spinal cord and cerebellum tRNA samples, the miRCURY LNA RT kit was used according to the manufacturer’s instructions (Qiagen, 339340, Hilden, Germany). Optional RNA spike-ins were also included in the reverse transcription reactions. cDNA samples were kept at −20 °C until further use.

For qPCR, GoTaq qPCR Master Mix was used according to the manufacturer’s instructions (Promega, A6001, Madison, WI, USA). cDNA samples were diluted 1:20 in nuclease-free water. Then, 96-well skirted PCR plates (Sarstedt, 72.1980.010, Nümbrecht, Germany) were used together with the following primers: UniSp6 (Qiagen, YP00203954, Hilden, Germany) and mmu-miR-223-3p (Qiagen, YP00205986, Hilden, Germany). qPCR was performed by the CFX96 Real-Time PCR Detection System (Bio-Rad, Hercules, CA, USA). Ct values produced at the end of the qPCR were used to quantify the miRNA levels in WT and WR samples. The differences in the expression levels were calculated using the 2^−ΔΔCt^ method with endogenous normalization to UniSp6.

### 2.5. Western Blotting

For protein isolation, cerebellum and cervical spinal cord tissues from WT and WR mice were homogenized in 1X RIPA lysis buffer (Cell Signaling, 9806S, Danvers, MA, USA) supplemented with Halt Protease Inhibitor Cocktail (Thermo Scientific, 1862209, Waltham, MA, USA; 10 µL mixture for 1 mg sample). After homogenization, samples were sonicated for 30 s; 1 cycle at 65% power. Then, samples were kept on ice for 30 min and centrifuged at 12,700 rpm for 15 min at 4 °C. The supernatants were transferred into new tubes and kept at −80 °C until further use. The concentrations of protein samples were determined using the Pierce BCA Protein Assay Kit, based on the manufacturer’s instructions (Thermo Fisher Scientific, 23227, Waltham, MA, USA). Then, 50–75 µg protein samples were denatured within 4X Laemmli buffer (Bio-Rad, 1610747, Hercules, CA, USA) either by the addition of DTT (Bio-Rad, 1610610, Hercules, CA, USA; for c-Caspase 1) or β-mercaptoethanol (Serva, 28625.01, Heidelberg, Germany; for the rest). Different denaturation processes were used for each antibody: CASP 1: 95 °C, 5 min; c-CASP 1: 100 °C, 10 min; GSDMD-FL, C-GSDMD, and NLRP3: room temperature, 30 min; IL-1β and IL-18: 100 °C, 5 min. Denatured protein samples were separated using 10–15% sodium dodecyl sulfate-polyacrylamide gel electrophoresis (SDS-PAGE). The Trans-Blot Turbo Transfer System (Bio-Rad, 690BR018169, Hercules, CA, USA) was used to transfer the SDS-PAGE gel to the 0.45 µm nitrocellulose membrane (Macherey-Nagel, 741280, Düren, Germany). Each blot was incubated with different blocking solutions for 1 h at room temperature: CASP 1, c-CASP 1, GSDMD-FL, NLRP3, IL-1β, and IL-18: 5% non-fat milk in Tris-buffered saline (TBS) containing 1% tween 20 (TBS-T); C-GSDMD: 5% BSA (Roth, 8076.2, Karlsruhe, Germany) in TBS-T; calnexin and actin: 1X ROTI-TBS (Carl Roth, A151.2, Karlsruhe, Germany). After blocking, membranes were incubated with primary antibodies at 4 °C overnight. Primary and secondary antibodies (Table 1) were diluted in the corresponding blocking solutions, except that the NLRP3 primary antibody was diluted in 5% BSA–TBS-T. The next day, membranes were washed and then incubated with the horse-radish-peroxidase (HRP)-conjugated secondary antibodies at room temperature for 1 h. After the washing steps, imaging of the membrane was performed using Clarity Western ECL Substrate (Bio-Rad, 1705060, Hercules, CA, USA). The chemiluminescent signal was captured using the Bio-Rad Chemiluminescence System (Bio-Rad, Universal Hood II, Hercules, CA, USA). Image Lab 6.1 (Bio-Rad, Hercules, CA, USA) was used to measure band intensities and all samples were normalized to either actin or calnexin blots as a loading control.

### 2.6. Immunofluorescence Staining

WT and WR mice were anesthetized with 10 mg/kg xylazine and 100 mg/kg ketamine in a saline solution. The animals were trans-cardiac perfused with 4% PFA (VWR Chemicals, 28794.295, Darmstadt, Germany), and the cervical parts of the spinal cords were removed. Cervical spinal cord tissues were transferred into 1.5 mL tubes, including 1 mL 4% PFA, and incubated at room temperature for 3 h with mild shaking. For the immunofluorescence staining, the collected tissues were washed 3 times with PBS and transferred into 30% sucrose (J.T. Baker, 0334, VWR Chemicals, Darmstadt, Germany) in PBS for cryoprotection. Tissues were incubated at 4 °C for at least 72 h in a 30% sucrose solution. Next, tissues were deep-frozen in isopentane at −45 °C for 5–10 s and were kept at −80 °C until further use.

For cryosectioning, tissues were embedded in the Tissue-Tek O.C.T. Compound (Sakura, 4583, Alphen aan den Rijn, The Netherlands), and 12 µm-thick sections were collected on Superfrost Plus Adhesion Microscope Slides (Epredia, J1800AMNZ, Portsmouth, NH, USA). The cervical spinal cord sections from p40 WT and WR mice were double-labeled using cellular markers (NeuN, Iba1, and GFAP) and NLRP3, c-CASP 1, and GSDMD-FL (Table 2). Firstly, sections were incubated with 0.3% Triton-X PBS solution at room temperature for 15 min. Then, sections were blocked at room temperature for 1 h in different blocking solutions, based on the primary antibody (Table 3; M.O.M blocking reagent, Vector Laboratories, MKB-2213-1, Bulingame, CA, USA; horse serum, Sigma-Aldrich, H1138, St. Louis, MO, USA; BSA, Roth, 8076.2, Karlsruhe, Germany). Then, sections were incubated with primary antibodies, first at room temperature for 1 h, then at 4 °C overnight. On the following day, slides were washed 3 times with PBS and incubated with corresponding fluorescent-labeled secondary antibodies in the dark at room temperature for 2 h (Table 2). Next, slides were washed 3 times with PBS and stained with 1 µg/mL Hoechst (Sigma-Aldrich, B1155, St Louis, MO, USA) in PBS for 20 min at room temperature. Finally, slides were washed 3 times with PBS and mounted with 24 × 40 mm coverslips by adding 2 drops of fluoroshield (Sigma-Aldrich, F6937, St Louis, MO, USA). Both primary and secondary antibodies were diluted in the blocking solutions. Control experiments were performed by the omission of primary antibodies. Images were captured using a Keyence BZ-X810 (Keyence Deutschland GmbH, Neu-Isenburg, Germany) fluorescent microscope and mean intensities were measured using ImageJ 2 2.9.0 (National Institutes of Health, Bethesda, MD, USA). Mean intensities measured from wild-type samples were averaged (Avg_WT_). Then, all of the mean intensity values, both from the wild-type and wobbler images, were divided by Avg_WT_ and multiplied by 100% to obtain percentage values. Colocalization analysis was performed using Imaris 10.1 (Oxford Instruments, Schlieren, Switzerland. Briefly, images from two different channels were segmented using the surface function, and then surface–surface colocalization was used to calculate the percentage of colocalized areas.

### 2.7. Statistical Analysis

Statistical analyses were performed using GraphPad Prism 9.4.1. All data were presented as mean ± standard error of the mean (SEM). Student’s *t*-test was used to determine the significance. For the miR-223-3p analysis of lasered spinal cord samples, 8 wild-type and 8 wobbler mice were used in triplicate for RT-qPCR. For the miR-223-3p analysis of spinal cord and cerebellum tissues, 4 wild type and 4 wobbler mice were used in triplicate for RT-qPCR. For Western blot analysis, 3–12 samples per group were used. For tissue immunostainings, 5 spinal cord slices were sectioned, and 10 (right and left ventral horns) images were taken. Each immunostaining was repeated for 3 animals per group. Any difference among experimental groups was accepted as significant in all evaluations when *p* < 0.05. Significances were depicted as follows: * *p* < 0.05; ** *p* < 0.01; *** *p* < 0.001; **** *p* < 0.0001.

## 3. Results

### 3.1. Wobbler Mice Demonstrated Increased Levels of miR-223-3p

First, we aimed to investigate miR-223-3p levels in the ventral horn of cervical spinal cord tissue in p40 wobbler mice. For this purpose, we stained the cervical spinal cord with cresyl violet and dissected the ventral horn using an LMD microscope (Figure 1A–C). After performing RT-qPCR, miR-223-3p expression was shown to be increased in the ventral horn of the wobbler mice (Figure 1D; *p* = 0.0010). To validate this upregulation, miRNAs were additionally isolated from the cervical spinal cord and cerebellum tissues of p40 wild-type and wobbler animals, and subsequently analyzed by RT-qPCR. The results showed that miR-223-3p was significantly upregulated both in the cervical part of the spinal cord (Figure 1E; *p* < 0.0001) and in the cerebellum (Figure 1F; *p* = 0.0003) of wobbler mice.

### 3.2. Increased NLRP3 Inflammasome Activation as a Sign of Pyroptotic Cell Death in the Wobbler Spinal Cord

**Figure 2:**
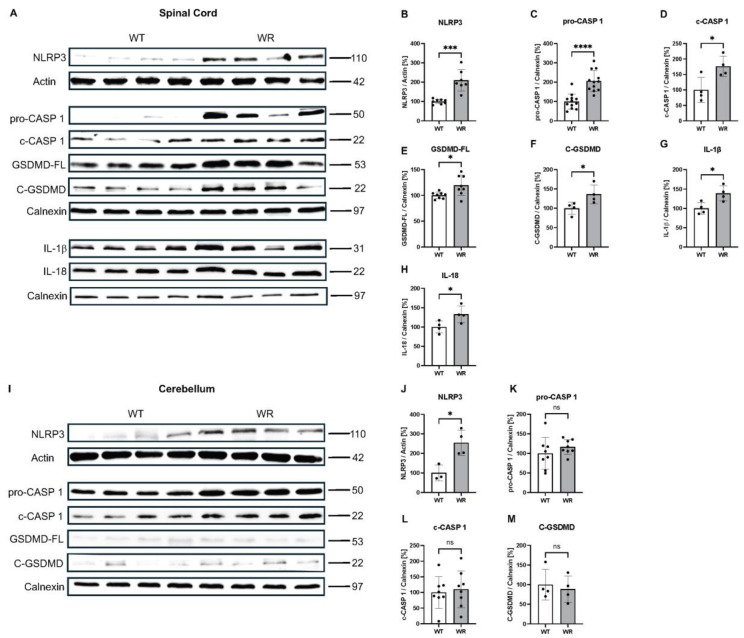
Increased levels of NLRP3, pro-CASP 1, c-CASP 1, GSDMD-FL, C-GSDMD, IL-1β, and IL-18 proteins revealed NLRP3 inflammasome activation and thus pyroptotic cell death in wobbler spinal cords, but not in the cerebellum, despite increased NLRP3 expression. (**A**) Western blot image and semi-quantitative analyses of (**B**) NLRP3, (**C**) pro-CASP 1, (**D**) c-CASP 1, (**E**) GSDMD-FL, (**F**) C-GSDMD, (**G**) IL-1β, and (**H**) IL-18 in cervical spinal cord tissues of p40 wild-type and wobbler mice. (**I**) Western blot image and quantitative analyses of (**J**) NLRP3, (**K**) pro-CASP 1, (**L**) c-CASP 1, and (**M**) C-GSDMD in cerebellum tissues of p40 wild-type and wobbler mice. Calnexin or actin was used as a loading control. Data are presented as mean ± SD, n varies between 4 and 12 per group. ns: not significant. * *p* < 0.05; *** *p* < 0.001; **** *p* < 0.0001.

Western blot analyses demonstrated a significant upregulation in the NLRP3 inflammasome components and pyroptotic cell death in the cervical spinal cord samples of the p40 wobbler mice (Figure 2A–H). The protein levels of NLRP3 (*p* = 0.0001) and pro-CASP 1 (*p* < 0.0001) were increased twofold in the wobbler spinal cord samples. Similarly, the amounts of c-CASP 1, GSDMD-FL, and C-GSDMD were increased by 76.3% (*p* = 0.0263), 19.5% (*p* = 0.0175), and 26.3% (*p* = 0.0448) in wobbler mice, respectively. As the downstream signaling cytokines of the NLRP3 inflammasome and pyroptotic cell death, IL-1β (*p* = 0.0201) and IL-18 (*p* = 0.0458) were found to be upregulated in the spinal cord of the p40 wobbler mice. Furthermore, the Western blot analyses of the p20 mice revealed no difference in the protein levels of NLRP3 (*p* = 0.1976), c-CASP 1 (*p* = 0.6298), or C-GSDMD (*p* = 0.6144) between wild-type and wobbler mice (Figure S1).

As an alternative to spinal cord tissue, we investigated the proteins from the cerebellum of p40 wild-type and wobbler mice by performing Western blot (Figure 2I–M). The Western blot analyses revealed no significant differences in the protein levels of pro-CASP 1 (*p* = 0.3194), c-CASP 1 (*p* = 0.7108), or C-GSDMD (*p* = 0.6717). The amount of GSDMD-FL was not analyzed due to the lower signal amounts; however, it was visible that there was no difference among the groups. The protein levels of NLRP3 (*p* = 0.0150) increased by approximately 155% in the cerebellum tissue of the wobbler mice.

### 3.3. Cell Type Specific Localization of NLRP3, CASP 1, and GSDMD in the Wobbler Spinal Cord

**Figure 3:**
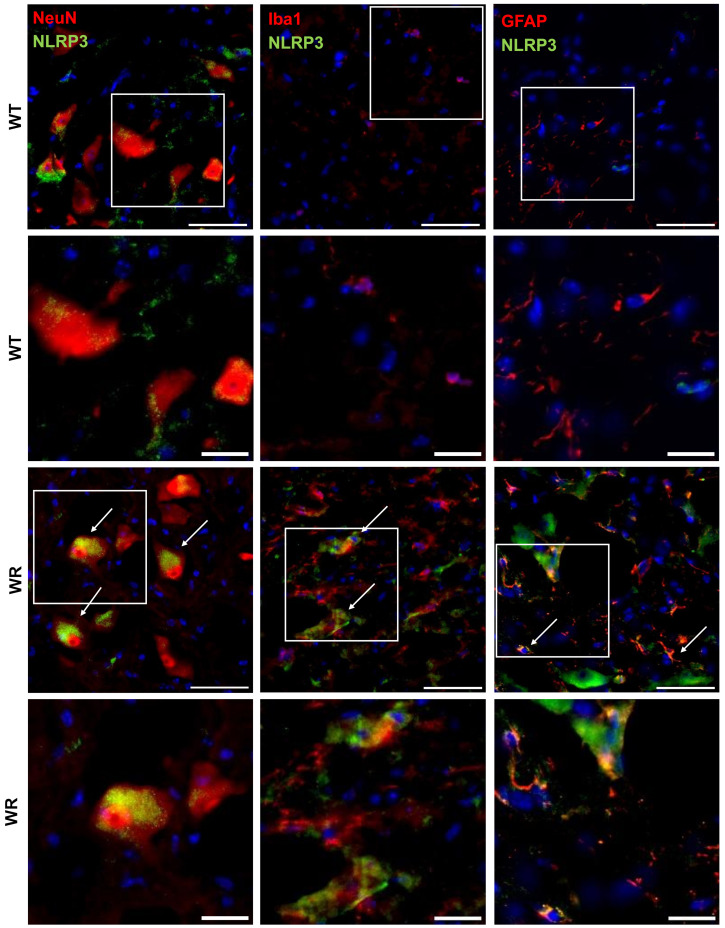
Representative images of double immunofluorescence staining for NLRP3 with NeuN, Iba1, or GFAP in the cervical spinal cord tissues of p40 wild-type and wobbler mice. NLRP3 was predominantly expressed in wobbler NeuN^+^ cells (motor neuronal cells), as well as in Iba1^+^ (microglia) and GFAP^+^ (astrocytes), which were in close proximity to motor neurons. Arrows indicate double-labeled cells. White frames indicate the areas of magnification. Scale: 50 µm; scale in the close-up images: 20 µm.

**Figure 4:**
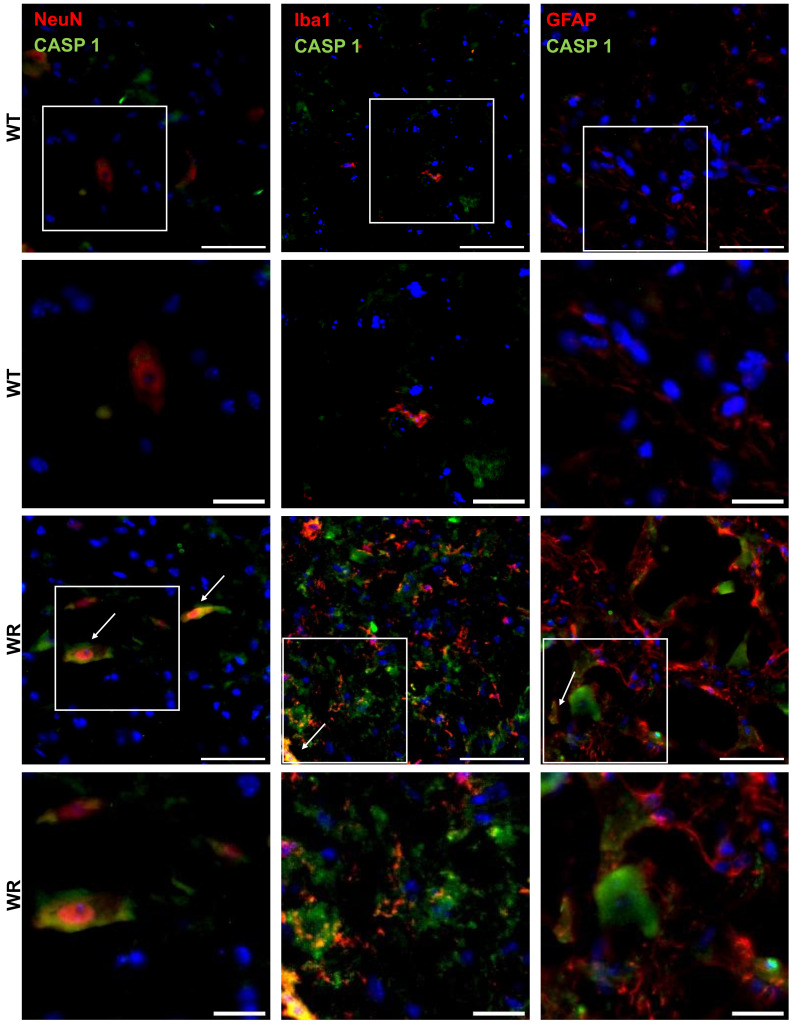
Representative images of double immunofluorescence staining for CASP 1 with NeuN, Iba1, or GFAP in the cervical spinal cord tissues of p40 wild-type and wobbler mice. CASP 1 was predominantly expressed in wobbler NeuN^+^ (motor neuronal cells), as well as in Iba1^+^ (microglia) and GFAP^+^ (astrocytes), which were in close proximity to motor neurons. Arrows indicate double-labeled cells. White frames indicate the areas of magnification. Scale: 50 µm; scale in the close-up images: 20 µm.

**Figure 5:**
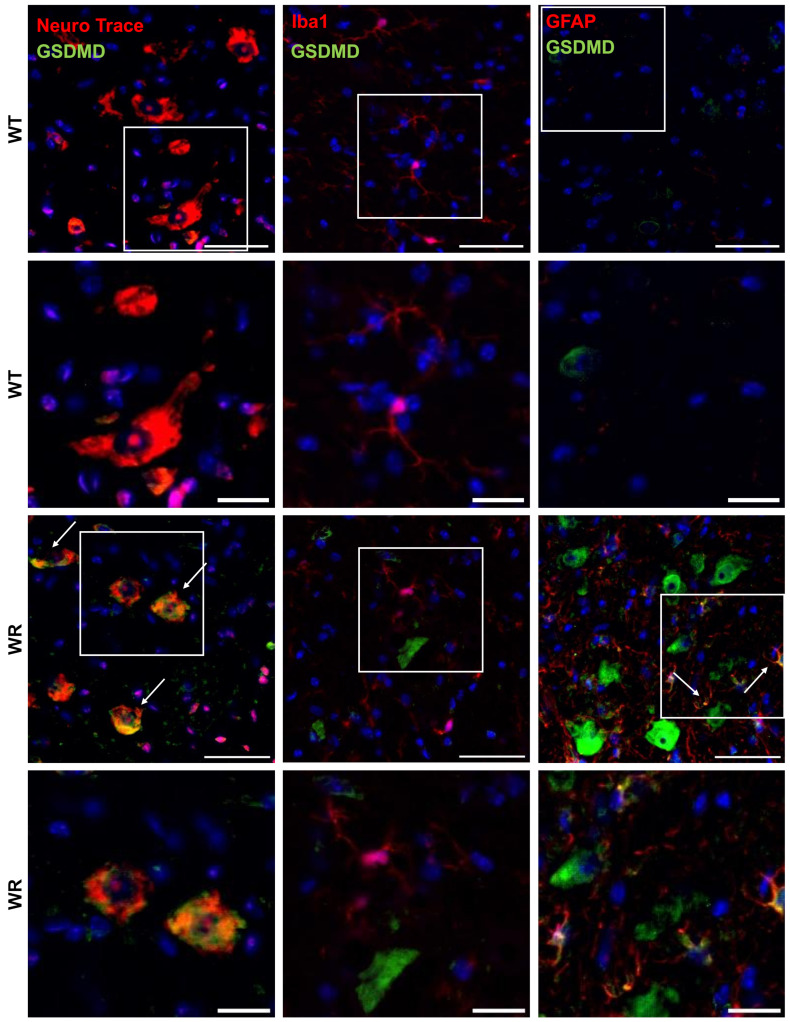
Representative images of double immunofluorescence staining for GSDMD with Neuro Trace, Iba1, or GFAP in the cervical spinal cord tissues of p40 wild-type and wobbler mice. GSDMD was predominantly expressed in wobbler NeuN+ (motor neuronal cells), as well as in GFAP^+^ (astrocytes), which were in close proximity to motor neurons. Arrows indicate double-labeled cells. White frames indicate the areas of magnification. Scale: 50 µm; scale in the close-up images: 20 µm.

After showing the upregulation of NLRP3, CASP 1, and GSDMD in the wobbler spinal cord tissue, we wanted to investigate the cell-type-specific localization of the aforementioned proteins in the cervical spinal cord. Therefore, double immunofluorescence staining was performed using spinal cord tissues of p40 wild-type and wobbler mice. We observed that significant upregulation of NLRP3 (Figure 3), CASP 1 (Figure 4), and GSDMD (Figure 5) in the wobbler spinal cord was mainly located in NeuN^+^ cells. Additionally, NLRP3, CASP 1, and GSDMD could be found to some extent in Iba1^+^ and GFAP^+^ cells in close proximity to neurons.

**Figure 6:**
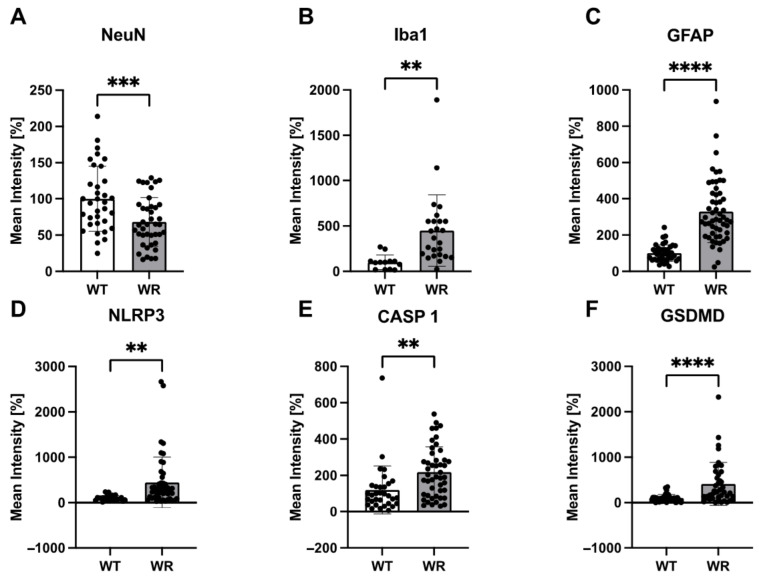
The statistical mean intensity analysis of (**A**) NeuN, (**B**) Iba1, (**C**) GFAP, (**D**) NLRP3, (**E**) CASP 1, and (**F**) GSDMD in the spinal cord of p40 wild-type and wobbler mice. Data are presented as mean ± SD, n is at least 30 per group. ** *p* < 0.01; *** *p* < 0.001; **** *p* < 0.0001.

Based on the statistical analyses performed by calculating the mean intensities of all images, motor neuron degeneration in the p40 wobbler spinal cord was indicated by decreased NeuN levels (*p* = 0.0008; Figure 6A). Furthermore, microgliosis and astrogliosis in the p40 wobbler spinal cord were demonstrated by increased levels of Iba1 (*p* = 0.0032; Figure 6B) and GFAP (*p* < 0.0001; Figure 6C). Additionally, NLRP3 (*p* = 0.0029; Figure 6D), CASP 1 (*p* = 0.0024; Figure 6E), and GSDMD (*p* < 0.0001; Figure 6F) were found to be significantly upregulated in the p40 wobbler spinal cord tissue. The analyses of double immunofluorescence staining regarding the colocalization of NLRP3, CASP 1, and GSDMD with cell-specific markers in spinal cord tissue revealed the colocalization of all proteins with all the investigated cell-specific markers, exclusively in wobbler mice (Table 4). In contrast, the wild-type mice exhibited no colocalization of NLRP3 signals with the microglial marker Iba1.

## 4. Discussion

ALS is the most prevalent motor neuron disease in adults, and apart from a few medications, there is currently no proper treatment to slow the symptoms down or halt the progression of the disease. Recent studies have identified mutations involved in the vesicular transport proteins in ALS patients. These genes include *Alsin 2* [36], *Alsin 8* [37], and *CHMP2B* [38]. In addition, ALS patients with mutations in the *Vps54* gene were found based on the Project MinE study (http://databrowser.projectmine.com, accessed on 5 March 2024). All these recent advances are evidence that the vesicular transport system is impaired in ALS pathology and therefore, the wobbler mouse as an sALS animal model has gained further importance. The main cause of the various symptoms in wobbler mice, from motor neuron degeneration at the cellular level to various symptoms at the organism level, is a point mutation in the *Vps54* gene. However, the exact cellular mechanisms through which this wobbler mutation in one of the proteins involved in vesicular transport contributes to motor neuron degeneration and in parallel causes various phenotypic changes are still unclear. In this study, using wobbler mice with a point mutation in the *Vps54* gene as an sALS animal model, we uncovered the activation of the NLRP3 inflammasome and thus pyroptotic cell death as another mechanism underlying motor neuron degeneration in the spinal motor neurons.

Many neurodegenerative diseases, including ALS, have been associated with the dysregulation of miRNAs. The first miRNA profiling study using spinal cords from sALS patients revealed a global miRNA downregulation [39]. In parallel, miRNA dysregulation was observed in the serum, cerebrospinal fluid, and leukocytes of ALS patients [40]. Similarly, several miRNAs such as miR-29b-3p and miR-375-3p were found to be dysregulated in the spinal cord and cerebellum tissues of wobbler mice [41,42]. Not surprisingly, considering that the wobbler mice have a 50% decrease in their mRNA levels [43], these data suggest that there might be a global RNA metabolism deficit in wobbler mice. In light of this information, in this study, we isolated miRNAs from the lasered ventral horn to examine miRNA differences in wild-type and wobbler mice. Of the different miRNAs examined, only miR-223-3p showed a significant upregulation in the lasered ventral horn of the p40 wobbler mice. Likewise, we demonstrated miR-223-3p upregulation in p40 wobbler spinal cord and cerebellum tissues. miR-223-3p is encoded by a gene located on the X chromosome, and its transcription is independent of any known gene [44]. miR-223-3p has been shown to be upregulated by the transcription factor NRF2 [45]. Unpublished data from our group demonstrated a significant increase in the mRNA level of *Nrf2* in the spinal cord samples. Therefore, the significant upregulation of miR-223-3p in the wobbler samples could be attributed to the upregulation of NRF2 levels in the wobbler mouse. It is also known that the increased levels of oxidative stress shown in p40 wobbler spinal cord [32] and spinal-cord-derived motor neurons [31] favors the upregulation of NRF2 in these samples. However, it is still undetermined which cellular mechanisms miR-223-3p upregulation affects in the wobbler mouse and what consequences it may have.

To investigate the implications of this significant miR-223-3p increase in wobbler mice in more detail, we focused our attention on the targets of miR-223-3p. On the one hand, miR-223-3p has been linked to the pro-inflammatory response. For example, in an animal model of multiple sclerosis, the upregulation of miR-223-3p was shown to increase inflammation [46,47,48]. Furthermore, it has been shown that IkappaB kinase α (IKKα), an inhibitory regulator of NFκB, is targeted by miR-223-3p, thereby inducing inflammation [49]. Moreover, in SOD1 mice, spleen-derived monocytes have been shown to upregulate miR-223-3p levels in association with the inflammatory response [50]. On the other hand, miR-223-3p has been associated with the anti-inflammatory response. For example, miR-223-3p upregulation was shown to reduce NLRP3 inflammasome activation by targeting NLRP3 itself [51]. Furthermore, miR-223-3p may target proteins in the NFκB pathway, such as TNF receptor-associated factor 6 (TRAF6) and TGF-beta activated kinase 1 binding protein 1 (TAB1), leading to the inhibition of NFκB translocation into the nucleus [52]. Additionally, it is known that Cullin 1 (CUL1a/b), which ubiquitinates IκB, can be targeted by miR-223-3p, resulting in the inhibition of NFκB translocation into the nucleus [52]. Moreover, the upregulation of miR-223-3p in neuronal regeneration experiments has been considered as an anti-inflammatory mechanism against neuronal degeneration [53,54]. Overall, miR-223-3p is predicted to (i) target proteins involved in anti-inflammatory pathways and (ii) target pro-inflammatory proteins but fail to degrade them adequately. Given the upregulation of *NFκB* [55] and intense inflammation shown by microgliosis and astrogliosis [5,56] in the spinal cord of wobbler mice, in addition to the dual effect of miR-223-3p on inflammation as explained above, it was inevitable to investigate NLRP3 inflammasome activation in this animal model.

NLRP3 is the most widely studied inflammasome complex among the known inflammasome types, and its role in several different neurodegenerative diseases has been investigated [57]. The post-mortem analysis from ALS patients revealed NLRP3 inflammasome activation in brain [58] and spinal cord [59] tissues. Likewise, blood samples [60] and skeletal muscle [61] of ALS patients showed the upregulation of NLRP3. Similarly, different animal models for ALS demonstrated NLRP3 inflammasome activation. For example, components of the NLRP3 inflammasome complex were found to be upregulated in the skeletal muscle [60], spinal cord [59], and brain [62,63] of SOD1 animal models. Moreover, NLRP3 inflammasome activation was detected in in vitro ALS models of TDP-43 [64] and C9Orf72 [65,66,67]. However, until now, NLRP3 inflammasome activation has not been investigated in wobbler mice, the only naturally occurring sALS animal model.

In this study, we revealed NLRP3 inflammasome activation in the spinal cord tissue of the wobbler mice, but not in the cerebellum. Several different DAMP molecules such as IL-1β, TNFα, HMGB1, TLR4, and TNFR have been shown to be upregulated [55,68,69,70,71,72,73], in addition to increased levels of *NFκB* [55,68] in wobbler spinal cord tissue, suggesting that the first step of the NLRP3 inflammasome activation has taken place. Normally, basal expressions of NLRP3 and IL-1β are not sufficient to induce NLRP3 formation [15], and only in the presence of activators such as elevated levels of ROS and mitochondrial damage can the NLRP3 inflammasome be activated. Since elevated levels of ROS [31,32] and mitochondrial damage [33] are present in the wobbler spinal cord tissue, the second step of NLRP3 inflammasome activation could also be triggered. In line with these findings, we demonstrated the upregulation of NLRP3, pro-CASP 1, and c-CASP 1 in the p40 wobbler spinal cord, but not in the p20 wobbler spinal cord. Furthermore, there was no significant change in ROS levels in the spinal cord tissue of the p20 wobbler mice [32], which may indicate that the NLRP3 inflammasome is not yet activated in wobbler mice at this age. Except for the upregulation of NLRP3, we could not detect any differences in pro-CASP 1 or c-CASP 1 levels in the cerebellum tissue among the p40 sample groups. Additionally, no significant difference was found when ROS levels in the cerebellum of the p40 wild-type and wobbler mice were compared [74]. The elevation of NLRP3 in the cerebellum could indicate the possible effect of NFκB on this gene; however, this increase alone does not guarantee inflammasome activation, which can also be inferred from unchanged levels of c-CASP 1 in the cerebellum. It should also be noted that the activation of other inflammasome complexes aside from NLRP3 was not examined in this study.

Pyroptotic cell death is an inevitable consequence of inflammasome activation and the cleavage of pro-CASP 1. It is also known that not all motor neurons in ALS degenerate through apoptosis [75]. Therefore, in this study, we showed the upregulation of GSDMD-FL and C-GSDMD, which indicated the cleavage of GSDMD-FL into C-GSDMD in the p40 wobbler spinal cord tissue, but not in the cerebellum. Furthermore, since there was no significant difference in C-GSDMD levels in the spinal cord tissue among the p20 mice, pyroptotic cell death is unexpected in this tissue at this stage. Pyroptotic cell death is only observed in p40 wobbler spinal cord, whereas in the wobbler cerebellum, activation of pro-CASP 1 does not occur; hence, the lack of pyroptotic cell death is not unusual. Furthermore, we investigated IL-1β and IL-18, being downstream cytokines of the NLRP3 inflammasome activation and pyroptotic cell death, and we showed the upregulation of these cytokines in the spinal cord of the p40 wobbler mice.

To investigate which cell types demonstrate NLRP3 activation and pyroptotic cell death, we performed double immunofluorescence staining using p40 spinal cord tissues. The results confirmed the motor neuron degeneration, microgliosis, and astrogliosis in the wobbler spinal cord. In a similar study, the early activation of microglia and astrocytes was demonstrated in the spinal cord of p6 wobbler mice [56]. Furthermore, we also confirmed the Western blot results and showed the upregulation of NLRP3, CASP 1, and GSDMD in the wobbler spinal cord in the immunofluorescence images. Moreover, the results from immunofluorescence staining indicated that NLRP3 activation and pyroptotic cell death were dominantly detected in motor neurons, in addition to being observed in microglia and astrocytes. Although classically NLRP3 inflammasome is primarily activated in microglia and astrocytes within the CNS, it has been observed that NLRP3 inflammasome activation can also take place in neurons in cases of neurodegeneration and nerve injury. For example, NLRP3, CASP 1, and IL-1β were found to colocalize in cortical neurons in ischemic stroke animal models [76,77]. Likewise, NLRP3, ASC, and CASP 1 were colocalized in neurons in animal models of traumatic brain injury and spinal cord injury [78,79]. Moreover, motor neurons in the spinal cord revealed increased expressions of NLRP3 and ASC proteins after peripheral nerve injury [80]. Furthermore, post-mortem tissues from Parkinson’s disease patients demonstrated upregulated *Nlrp3* expression in dopaminergic neurons [81]. The colocalization of NLRP3, CASP 1, and GSDMD within neurons was also shown in the spinal cord of SOD1 animal model [82]. An additional study on ALS showed that the NLRP3 inflammasome is mainly involved in astrocytes rather than microglia, although motor neurons were not examined in this study [59]. In summary, it is interesting to consider the possibility that wobbler motor neurons may use the inflammasome structure to sense intracellular triggers such as elevated levels of ROS [31,32], mitochondrial damage [33], and misfolded proteins [83], and then release cytokines to activate the neighboring astrocytes and microglia, enhancing chronic neuroinflammation in the wobbler spinal cord. Furthermore, although these intracellular triggers have not been investigated so far in the wobbler cerebellum, since we do not observe NLRP3 activation and pyroptotic cell death in this tissue, we can speculate that these activator molecules are preferentially available in the spinal cord. Nevertheless, it should also be noted that non-cell autonomous cell death may also trigger motor neuron degeneration in the spinal cord in the first place [84]. It is also likely that these cellular processes occur at the earliest stages of motor neuron degeneration in the wobbler mouse and can exacerbate ALS pathology.

**Figure 7:**
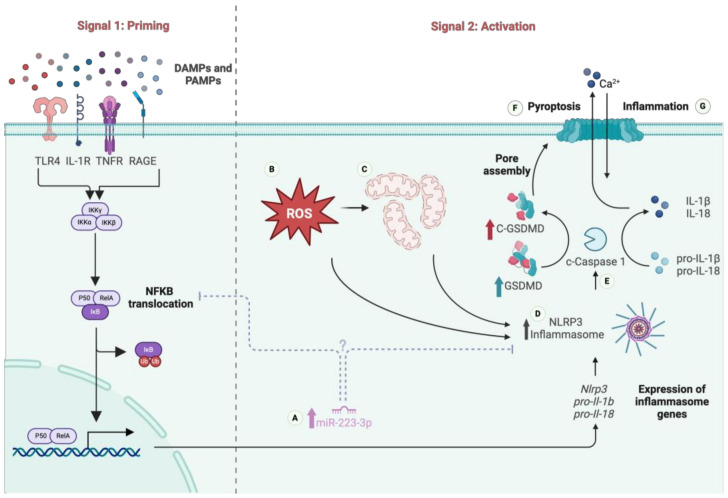
Activation of NLRP3 inflammasome and pyroptotic cell death in motor neurons of the wobbler spinal cord. During the priming step, DAMP and PAMP molecules activate several different receptors such as TLR4, IL-1R, TNFR, and RAGE. This activation leads to ubiquitination and thereby degradation of IκB, the inhibitor of NFκB. As a consequence, P50 and RelA subunits of NFκB can be translocated into the nucleus, where they can upregulate the expression of inflammasome genes such as Nlrp3, pro-Il-1b, and pro-Il-18. (**A**) Increasing levels of miR-223-3p has been shown in the lasered ventral horn and cervical spinal cord of the wobbler mouse. However, it is unclear whether miR-223-3p targets NLRP3 or an alternative protein(s) in the NFκB translocation pathway in wobbler mice. (**B**) Elevated levels of ROS and (**C**) mitochondrial fragmentation in the spinal cord of the wobbler motor neurons trigger (**D**) NLRP3 inflammasome formation, leading to (**E**) auto-cleavage of pro-CASP 1 into c-CASP 1. On one hand, c-CASP 1 is able to cleave pro-IL-1β and pro-IL-18 into their mature forms, and on the other hand, it can cleave GSDMD protein. N-GSDMD subunits can translocate into the cellular membrane and form pores, which cause membrane rupture and eventually (**F**) pyroptotic motor neuronal death. Through these pores, mature cytokines can be released, leading to (**G**) inflammation for the neighboring cells. This image was created with BioRender.com.

Based on these findings, it can be clearly stated that NLRP3 inflammasome activation and subsequent pyroptotic cell death is one of the mechanisms contributing to motor neuron degeneration in the wobbler spinal cord (Figure 7). Further research is needed to understand whether there is a direct relationship between miR-223-3p and NLRP3 inflammasome activation in wobbler spinal cord samples. These investigations can be started by crossing a wobbler mouse with an NLRP3-knockout mouse. Although it is not certain whether miR-223-3p directly inhibits NLRP3 or inhibitors of the NFκB pathway, it is also possible that these two events occur simultaneously and shift towards a pro-inflammatory direction in wobbler mice. Additionally, NLRP3 activation and pyroptotic cell death should be kept in mind when developing future therapies to prevent motor neuron death in ALS. In conclusion, this is the first study to show that NLRP3 inflammasome activation and pyroptotic cell death in the wobbler spinal cord are observed in motor neurons, which can further exacerbate motor neuron degeneration seen in wobbler mice.

## 5. Conclusions

Overall, this study reveals that NLRP3 inflammasome activation and pyroptotic cell death are involved in the spinal cord, but not in the cerebellum of wobbler mice. These cellular mechanisms take place especially in motor neurons and lead to the exacerbation of disease progression. Additionally, the effect of miR-223-3p on the regulation of NLRP3 inflammasome activation remains elusive. Considering motor neuron degeneration in ALS, therapeutic approaches targeting NLRP3 inflammasome activation and pyroptotic cell death are highly likely to be promising. 

## Figures and Tables

**Table 1:** Antibody list for Western blotting.

Antibody	Company	Catalogue Number	Dilution
Anti-Actin	Sigma-Aldrich, St Louis, MO, USA	A5060	1:250
Anti-c-Caspase 1	Cell Signaling, Danvers, MA, USA	89332	1:1000
Anti-C-GSDMD	Abcam, Cambridge, UK	255603	1:1000
Anti-Calnexin	Novus Biologicals, Centennial, CO, USA	NB100-1965	1:1000
Anti-Caspase 1 (p45)	Adipogen, Liestal, CH	AG-20B-0042	1 µg/mL
Anti-Catalase	Santa Cruz, St Louis, MO, USA	SC-271803	1:200
Anti-GSDMD-FL	Abcam, Cambridge, UK	219800	1:2000
Anti-IL-1β	Protein Tech, Sankt Leon-Rot, Germany	26048-1-AP	1:1000
Anti-IL-18	Protein Tech, Sankt Leon-Rot, Germany	10663-1-AP	1:1000
Anti-NLRP3	Cell Signaling, Danvers, MA, USA	15101	1:1000
Anti-Mouse HRP Secondary	VectorLabs, Bulingame, CA, USA	VEC-PI-2000-1	1:10,000
Anti-Rabbit HRP Secondary	VectorLabs, Bulingame, CA, USA	VEC-PI-1000-1	1:10,000

**Table 2:** Antibody list for immunofluorescence staining.

Antibody	Company	Catalogue Number	Dilution
Anti-Chicken AF488	Invitrogen, Carlsbad, CA, USA	A-32931	1:400
Anti-Chicken AF568	Invitrogen, Carlsbad, CA, USA	A-11041	1:200
Anti-Goat AF488	Abcam, Cambridge, UK	150129	1:100
Anti-Goat AF594	Invitrogen, Carlsbad, CA, USA	A-11058	1:400
Anti-Goat FITC	Abcam, Cambridge, UK	Ab6881	1:400
Anti-Mouse AF488	Invitrogen, Carlsbad, CA, USA	A-11001	1:200
Anti-Mouse AF594	Invitrogen, Carlsbad, CA, USA	A-32744	1:400
Anti-Mouse TRITC	Sigma-Aldrich, St Louis, MO, USA	T5393	1:200
Anti-Rabbit AF488	Invitrogen, Carlsbad, CA, USA	A-11008	1:500
Anti-Rabbit AF488	Invitrogen, Carlsbad, CA, USA	A-21206	1:500
Anti-Rabbit TRITC	Sigma-Aldrich, St Louis, MO, USA	T6778	1:200
Anti-Rat AF594	Invitrogen, Carlsbad, CA, USA	A-11007	1:200
Anti-Caspase 1 (p20 & p45)	Adipogen, Liestal, Switzerland	AG-20B-0042	1:200
Anti-CD68	Bio-Rad, Hercules, CA, USA	MCA1957T	1:200
Anti-GFAP	Abcam, Cambridge, UK	4674	1:1000
Anti-GSDMD	Protein Tech, Sankt Leon-Rot, Germany	20770-1-AP	1:100
Anti-Iba1	Wako, Minneapolis	19741	1:1000
Anti-NeuN	Abcam, Cambridge, UK	177487	1:500
Anti-NLRP3	RND Systems, Minneapolis, MN, USA	MAB7578	5 µg/mL

**Table 3:** Blocking solutions and antibody diluents required for immunofluorescence staining.

Primary Antibody	Blocking Solution	Antibody Diluent
Caspase 1 (p20 and p45)	M.O.M blocking reagent (1 h) + 5% horse serum (1 h)	1% BSA, 0.3% Triton-X
GSDMD	5% BSA	1% BSA, 0.3% Triton-X
NLRP3	5% horse serum	1% BSA, 1% donkey, 0.3% Triton-X

**Table 4:** Colocalization analyses showing the percentages of colocalized areas of NLRP3, CASP 1, and GSDMD with NeuN (neurons), Iba1 (microglia), and GFAP (astrocytes) in p40 wild-type and wobbler spinal cord tissues.

Colocalization [%]	NeuN/Neuro Trace		Iba1		GFAP	
	WT	WR	WT	WR	WT	WR
NLRP3	33.67	61.19	0.000	51.30	3.780	19.59
CASP 1	71.79	79.25	37.98	60.07	6.750	31.02
GSDMD	4.600	64.36	46.81	9.150	6.570	35.11

## Data Availability

The data presented in this study will be provided by the corresponding author upon request.

## Supplementary Materials

The following supporting information can be downloaded at: https://www.mdpi.com/article/10.3390/cells13120995/s1, Figure S1: Protein levels of NLRP3, c-CASP 1, and C-GSDMD revealed no NLRP3 inflammasome activation and pyroptotic cell death in the spinal cord tissues of p20 wobbler mice. (**A**) Western blot image and semi-quantitative analyses of (**B**) NLRP3, (**C**) c-CASP 1, and (**D**) C-GSDMD in cervical spinal cord tissues of p20 wild-type and wobbler mice. Calnexin or actin was used as a loading control. Data are presented as mean ± SD, n varies between 3–4 per group. ns: not significant.
