# Supplementary material for: Elevated NLRP3 Inflammasome Activation Is Associated with Motor Neuron Degeneration in ALS
Source: Cells. 2024 Jun 7;13(12):995. doi: 10.3390/cells13120995 (PMC11202041; doi:10.3390/cells13120995)
Supplement: Supplementary file 1 [file cells-13-00995-s001.zip › cells-2991519-supplementary.pdf]

Elevated NLRP3 Inflammasome Activation is Associated with Motor Neuron Degeneration in ALS

Hilal Cihankaya [1,2], Verian Bader [3], Konstanze F. Winklhofer [2,3], Matthias Vorgerd [4], Johann Matschke [5], Sarah Stahlke [1], Carsten Theiss [1,2] and Veronika Matschke [1,2,*]

1 Department of Cytology, Institute of Anatomy, Ruhr-University Bochum, 44801, Germany hilalcihankaya@gmail.com (H.C.), sarah.stahlke@rub.de (S.S.), carsten.theiss@rub.de (C.T.), veronika.matschke@rub.de (V.M.)

2 International Graduate School of Neuroscience (IGSN), Ruhr-University Bochum, 44801, Germany

3 Department of Molecular Cell Biology, Institute of Biochemistry and Pathobiochemistry, Medical Faculty, Ruhr University Bochum, D-44801 Bochum, Germany verian.bader@rub.de (V.B.), konstanze.winklhofer@rub.de (K.F.W.)

4 Department of Neurology, Heimer Institute for Muscle Research, University Hospital Bergmannsheil, Ruhr-University Bochum, Buerkle-de-la-Camp-Platz 1, 44789 Bochum, Germany matthias.vorgerd@bergmannsheil.de (M.V.)

5 Institute of Cell Biology (Cancer Research), University Hospital Essen, University of Duisburg-Essen, 45147, Germany johann.matschke@uni-due.de (J. M.)

* Correspondence: veronika.matschke@rub.de; Tel.: +49 234 32 25018

Supplementary Figure S1

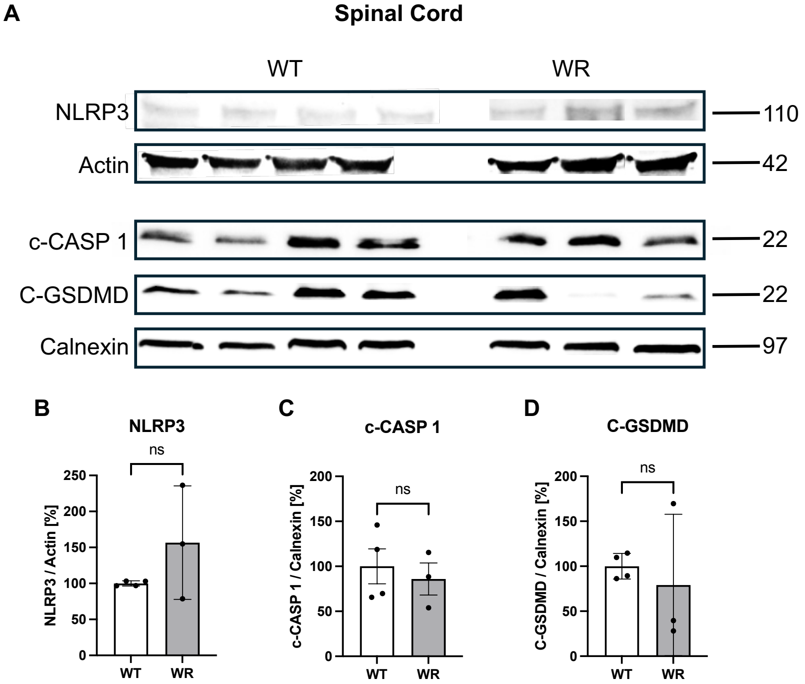

Figure S1. Protein levels of NLRP3, c-CASP 1, and C-GSDMD revealed no NLRP3 inflammasome activation and pyroptotic cell death in the spinal cord tissues of p20 wobbler mice. A) Western blot image and semi-quantitative analyses of B) NLRP3, C) c-CASP 1, and D) C-GSDMD in cervical spinal cord tissues of p20 wild-type and wobbler mice. Calnexin or actin was used as a loading control. Data are presented as mean ± SD, n varies between 3-4 per group. ns: not significant.
